# Efficacy of Whole-Brain Radiotherapy Plus Simultaneous Integrated Boost (SIB-WBRT) for Lung Cancer Brain Metastases

**DOI:** 10.7150/jca.95804

**Published:** 2024-07-02

**Authors:** Qian Bi, Jing Shen, Pengyu Li, Yuhao Zeng, Xin Lian, Fuquan Zhang

**Affiliations:** 1 Department of Radiation Oncology, State Key Laboratory of Complex Severe and Rare Diseases, Peking Union Medical College Hospital, Chinese Academy of Medical Sciences and Peking Union Medical College, Beijing, China.; 2 Department of General Surgery, Peking Union Medical College Hospital (PUMCH), Peking Union Medical College and Chinese Academy of Medical Sciences, Beijing, China; 3 Department of Internal Medicine, Cleveland Clinic, Akron General, Akron, OH, USA.

**Keywords:** brain metastases, simultaneous integrated boost, simultaneous integrated boost whole-brain radiation therapy, whole-brain radiation therapy

## Abstract

**Objective:** To investigate the outcomes of SIB-WBRT in patients with brain metastases and analyze the impact of some factors on prognosis.

**Materials and Methods:** This single-arm retrospective study analyzed patients with brain metastases who were treated with SIB-WBRT at Peking Union Medical College Hospital from September 2015 to December 2021. The primary endpoint was intracranial progression free survival (iPFS). Secondary endpoints included overall survival (OS), intracranial new foci, and tumor control. The Kaplan-Meier method was then used to depict and estimate iPFS, OS, intracranial neoplasia, and tumor control. Finally, the Cox model was used to analyze the association between some relevant factors and outcomes.

**Results:** A total of 107 patients were included and the median iPFS in these patients treated with SIB-WBRT was 13.4 (95% CI: 4.2-22.6) months, with 68.0% (95% CI: 57.4%-78.6%) and 50.8% (95% CI: 38.3%-63.3%) iPFS at 6- and 12-months. The median local control was 37.6 (95% CI: 28.3-46.8) months, with local control rates of 84.3% (95% CI: 80.6%-88.0%) and 73.3% (95% CI: 68.2%-78.4%) at 6- and 12-months. The median time to appearance of new intracranial foci was 17.4 (95% CI: 14.1-20.8) months, and the 6- and 12-month control rates were 74.5% (95% CI: 64.5%-84.5%) and 61.5% (95% CI: 49.0%-74.0%). The number of brain metastases in patients before treatment was significantly associated with iPFS (HR=0.4, 95% CI: 0.2-0.973, *P*=0.043).

**Conclusions:** The iPFS, local control, and intracranial new foci of patients with brain metastases after treatment with SIB-WBRT were acceptable. In addition, the number of brain metastases in patients before treatment may be associated with iPFS.

## Introduction

Brain metastasis is a prevalent and consequential issue encountered by numerous patients afflicted with malignant tumors 1. Whole-brain radiotherapy is a commonly used treatment for brain metastases, which plays a very important role in controlling brain metastases and improving the symptoms of cancer patients 2. However, conventional whole-brain radiotherapy is insufficient for effective control of brain metastases due to low doses.

In order to overcome these limitations and improve the therapeutic efficacy, Whole-Brain Radiotherapy Plus Simultaneous Integrated Boost (SIB-WBRT) has emerged as a new radiotherapy strategy 3. In SIB-WBRT, whole-brain irradiation can have a certain inhibitory effect on potential metastatic foci, and simultaneous booster in the lesion area has the potential to enhance the efficacy of local control for metastatic sites. Although theoretically there are certain benefits of SIB-WBRT, currently various medical institutions have not reached a unanimous conclusion on issues such as the exact prescribed dose and indications for SIB-WBRT, and meanwhile, high-quality clinical research data were lacking 4.

Therefore, we retrospectively analyzed the survival, local control, and new lesion in patients with brain metastases who underwent treatment with SIB-WBRT at Peking Union Medical College Hospital, aiming to provide some data support for the efficacy of SIB-WBRT. At the same time, we also analyzed data from similar studies that have been published internationally to more comprehensively assess the efficacy of this treatment strategy.

## Methods

### Patient enrollment

This study was a retrospective study. The study subjects were lung cancer patients with brain metastases who received SIB-WBRT treatment at the Department of Radiation Oncology, Peking Union Medical College Hospital from September 2015 to December 2021. Patients' inclusion criteria were:

(1) pathologically confirmed lung cancer and confirmed brain metastases by imaging;

(2) brain metastasis radiotherapy using SIB-WBRT;

(3) completion of the entire course of radiotherapy;

(4) at least one post-treatment imaging examination available for evaluating treatment efficacy. Patients' exclusion criteria were:

(1) no follow-up results (the patient did not undergo a follow-up examination or there was no relevant imaging data to evaluate the efficacy after treatment).

This study has been approved by the Ethics Review Committee of Peking Union Medical College Hospital (Approval number: S-K1982).

### Data collection

Relevant data of patients were obtained from the electronic medical record system of Peking Union Medical College Hospital. The following data were collected:

(1) clinical data: including primary disease, pathological type, molecular pathology, extracranial disease progression, number and volume of metastatic tumors, radiotherapy technique and dose fractionation, concurrent medication, etc.;

(2) general data: including general information such as gender, age, etc.;

(3) follow-up data: including treatment response of intracranial lesions (lesion control and appearance of new lesions), survival status, follow-up time, etc.

### Radiation therapy method

6MV X-ray linear accelerator was used for radiation therapy including volumetric modulated arc therapy (VMAT) technique, fixed field-intensity modulated radiotherapy (FF-IMRT) technique or TOMO Therapy (Tomo) technique. Thermoplastic mesh was used for patient immobilization, and 2-3mm CT slices were used for simulation and positioning. The positioning image was fused with enhanced MRI for delineation of target areas and organs at risk (OARs). The clinical target volume (CTV) was defined as the entire brain tissue, and the gross tumor volume (GTV) was defined as the visible tumor area on MRI images. CTV was expanded by 2-3mm to form the planning target volume (PTV). The prescribed dose at the GTV was 56-60Gy, and treatment plans were designed using Tomo, Eclipse or Monaco treatment planning systems. The prescribed dose at the CTV was 40Gy, and both were completed within 20 fractions, with 5 treatments per week. The volume dose limits for OARs were: ≤8Gy for 1% of the lens, and ≤54Gy for 0.03cc of the brainstem.

### Patient follow-up

All patients were followed up one month after treatment. Then it is recommended to follow up outpatient patients every three months. Enhanced MRI of the head is used for assessment of the therapeutic efficacy of brain metastases radiotherapy, based on the Response Assessment in Neuro-Oncology (RANO) criteria, by experienced radiation oncologists 5. Safety assessment was performed in the entire cohort, including radiation necrosis, hematology, and biochemical parameters. Adverse events were graded according to the Common Terminology Criteria for Adverse Events (Version 5.0) of the National Cancer Institute 6.

### Follow-up endpoints

The primary endpoint was intracranial progression-free survival (iPFS), defined as the time from the end of radiotherapy to intracranial radiographic progression or death 7. Radiographic progression includes uncontrolled recurrence of lesions or the appearance of new intracranial lesions 8.

Secondary endpoints included overall survival (OS), tumor progression time, and time to new intracranial lesions. OS was defined as the time from the end of radiotherapy to death or the last follow-up 9. Tumor progression time was defined as the time from the end of radiotherapy to the detection of tumor recurrence or the last follow-up. Time to new intracranial lesions was defined as the time from the end of radiotherapy to the detection of new lesions or the last follow-up.

### Statistical analysis

Based on chart review, 107 patients met the inclusion criteria. Kaplan-Meier method was used to depict and estimate iPFS, OS, the incidence of new intracranial lesions, and tumor control. If a patient had multiple lesions, multiple lesions data were analyzed as data basic points for local control. The Cox model was used to analyze the correlation between relevant factors (gender, age, pathological type of lung cancer, tumor volume, number of tumors, control of primary lesions during brain metastases, targeted therapy during brain metastases, and proportion of tumors in the whole brain) and outcomes, and both univariable and multivariable analyses were conducted. All factors were included in the multivariable analysis regardless of statistical significance in the univariable analysis. Categorical variables were compared using Chi-square or Fisher's exact test. All statistical analyses were performed using SPSS version 27.0 (IBM Corp, New York, USA), GraphPad Prism 8, and SAS 9.4 (SAS Institute Inc, Cary, NC). The significance level was set at a two-sided *P*-value of <0.05^10^.

## Results

### Patient baseline clinical characteristics

A total of 107 patients were included in this study, with a total of 776 brain metastases. Among these 107 patients, there were 63 males (58.9%) and 44 females (41.1%). In terms of age, 48 cases (44.9%) were below 60 years old and 59 cases (55.1%) were 60 years old or above. Regarding tumor pathology, there were 41 cases (38.3%) of small cell lung cancer and 66 cases (61.7%) of non-small cell lung cancer. In terms of pre-treatment conditions, 4 patients (3.7%) had well-controlled primary lesions during head radiotherapy, while 103 patients (96.3%) had poorly controlled primary lesions during head radiotherapy. Additionally, 52 patients (48.6%) received targeted therapy during the course of brain metastasis, while 55 patients (51.4%) did not receive targeted therapy. The median tumor volume at the time of radiation treatment planning was 8.4 cm^3^ (range: 0.4-73.3 cm^3^) 11. 18 patients (16.8%) had more than 10 brain metastases, while 89 patients (83.2%) had 10 or fewer brain metastases. The median value of the average dose received by the entire patient cohort in the brain was 43.0 Gy (range: 31.4-53.3 Gy). The median value of the percentage of brain metastases in the entire brain tissue was 0.6% (range: 0.03%-6.1%). Detailed data can be found in Table 1.

### Follow-up status

In these 107 patients, 53 patients experienced intracranial progression, with a median iPFS of 13.4 months (95% CI: 4.2-22.6). The rates of intracranial progression-free survival at 6 and 12 months post-treatment were 68.0% (95% CI: 57.4-78.6) and 50.8 (95%CI: 38.3-63.3), respectively 12. Additionally, 53 patients experienced intracranial progression within 0.6-45.7 months after radiotherapy.

Overall, the median overall survival for these 107 patients was 15.7 months (95% CI: 10.4-21.1%). Survival rates at 6, 12, and 24 months were 79.3% (95%CI: 71.5-87.1), 58.9% (95%CI: 49.3-68.5), and 35.9% (95%CI: 25.9-45.9), respectively.

These 107 patients had a total of 776 brain metastatic lesions, with 638 lesions still under effective control at the last follow-up, resulting in a local control rate of 82.2% after radiotherapy. The median progression time of metastatic lesions was 37.6 months (95% CI: 28.3-46.8). Control rates at 6 and 12 months were 84.3(95%CI: 80.6-88.0) and 73.3(95%CI: 68.2-78.4), respectively. Additionally, 138 lesions progressed within 0.9-46.6 months after radiotherapy.

**Figure 1:**
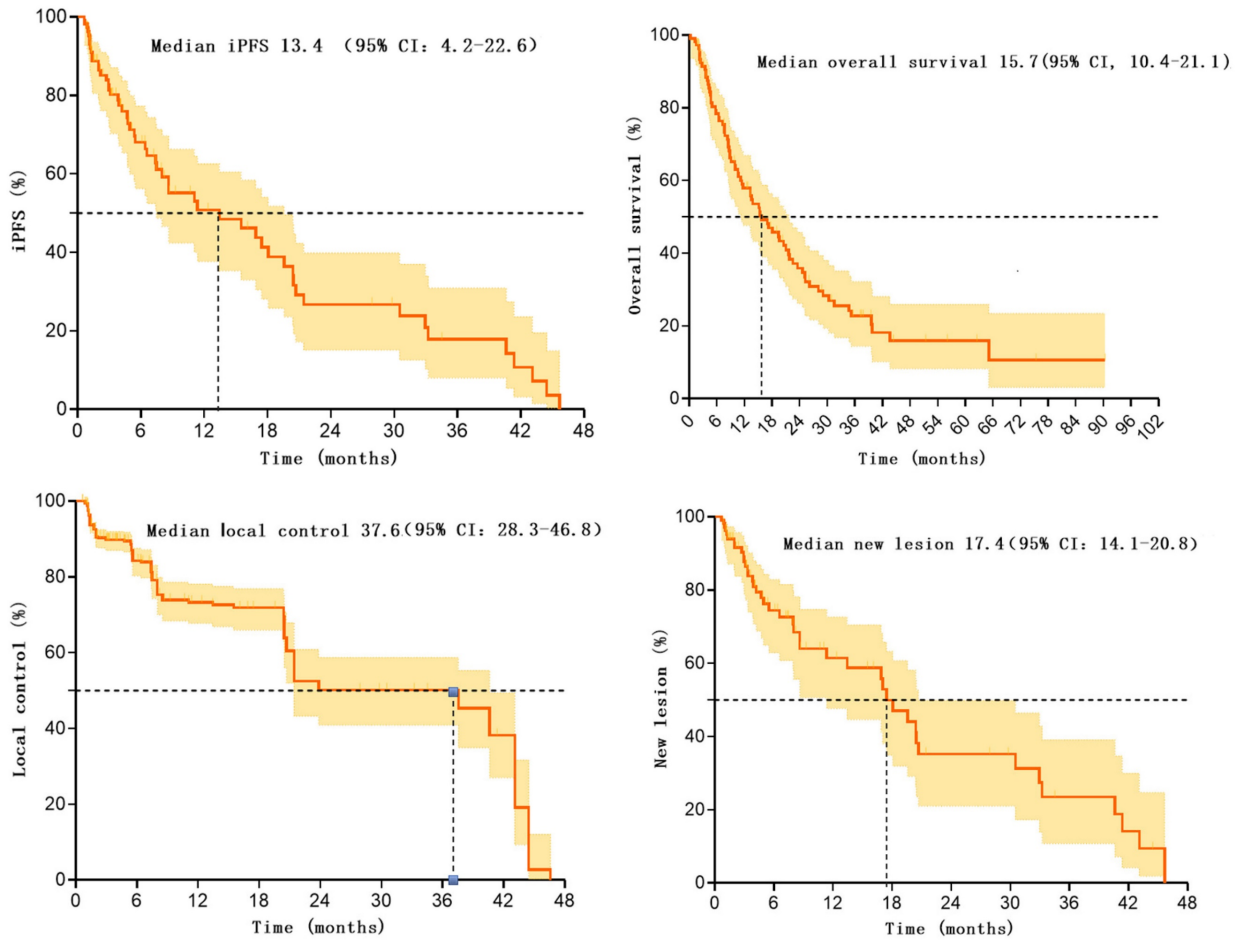
Kaplan-Meier survival curve for patients after SIB-WBRT.

Furthermore, among these 107 patients, 42 patients developed new intracranial lesions during follow-up, resulting in an incidence rate of 39.3%. The median time to the occurrence of new lesions was 17.4 months (95% CI: 14.1-20.8). The rates of no new brain metastases at 6 and 12 months were 74.5 (95%CI: 64.5-84.5) and 61.5 (95%CI: 49.0-74.0), respectively. There were 42 patients who developed new lesions within 0.7-45.7 months after radiotherapy. Please refer to Table 2 and Figure 1 for more details.

### Radiation necrosis

No cases of radiation necrosis were observed in the follow-up results of this patient group.

### Univariable and multivariable analysis

Univariable analysis using the Cox proportional hazards regression model revealed no significant predictors for iPFS. Multivariable analysis using the Cox proportional hazards regression model showed that the number of tumors was significantly associated with iPFS. In conclusion, the number of tumors is an independent prognostic factor for iPFS. Refer to Table 3 for details.

## Discussion

This study is about SIB-WBRT with a high number of patients, and the results of the related data can well fill the gap in this field. In this single-center retrospective study, we summarized the efficacy of using SIB-WBRT in controlling intracranial metastases in patients with brain metastases. The median value of the primary endpoint, iPFS, was 13.4 (95% CI: 4.2-22.6) months. The occurrence of radionecrosis was not observed in any of the patients, indicating that SIB-WBRT is safe and reliable. After further analysis, it was found that the number of brain metastases in patients before treatment might be an independent prognostic factor for iPFS 13-16.

SIB-WBRT does result in an increase in metastatic control compared to the WBRT regimen. Regarding the efficacy of WBRT, Ge et al. designed a study which included a total of 72 patients with brain metastases from lung cancer, and 38 patients were treated with WBRT (40Gy/20 fractions), and the 1-year control rate of intracranial lesions in this group was 41.6%, which was significantly lower than that of the other group of patients who were treated with SIB-WBRT (56-60Gy/20 fractions) (1-year control rate of 75.9%, P=0.049) 17, 18. Similar results were obtained in the multicenter study by Casanova et al. and RTOG9508, with 1-year control rates of 75.2% and 82.0% in patients in the SIB-WBRT group 19, 20. The SIB-WBRT results of these articles are consistent with the results of the data we obtained. We believe that the higher local control rate brought about by SIB-WBRT may be due to the fact that this technique targeted to increase the dose of tumor irradiated, thus controlling the growth of the metastases in the brain better than the WBRT regimen, but this conclusion still needs to be further confirmed by carrying out a relevant RCT study in the end.

SIB-WBRT has advantages over localized brain metastasis irradiation regimens in reducing the incidence of new lesions. Localized brain metastasis irradiation is more likely to result in the development of new brain metastases in patients with brain metastases treated with this regimen because no prophylactic irradiation of other normal brain tissue is performed. A 2013 retrospective study from the U.S. compared the efficacy of three modalities of radiation therapy for brain metastases, SIB-WBRT, WBRT, and localized brain metastasis irradiation. A total of 92 patients were analyzed in this study (75 Gy/10-15 fractions in the SIB-WBRT group, 30-37.5 Gy/10-15 fractions in the WBRT group, and 30 Gy/5 fractions in the localized brain metastasis irradiation group), and the analyzed results showed that the rate of new brain metastatic lesions in the SIB-WBRT group was significantly lower than that in the localized brain metastasis irradiation group (18% vs. 33%) 15, 16, 21, 22. Our data showed similar results, which are longer than that of other non-whole-brain irradiation (e.g., SRS) previously reported in the literature 23. One possible explanation is that SIB-WBRT has a wider irradiation range and can radiate entire brain tissue, including potentially occult lesions. This is important for controlling potentially tiny lesions or lesion residues. In contrast, localized irradiation or SRS usually targets only specific lesions for treatment and fails to cover the entire brain tissue. This may result in a failure to control underlying microscopic lesions, thereby increasing the risk of new lesions.

The efficacy of SIB-WBRT has been reported in several studies. 2021 Radiotherapists from Sichuan, China, published a study based on data from patients with brain metastases at their center, in which they included data from 37 patients treated with SIB-WBRT, and reported after retrospective analysis that the median iPFS of this group of patients was 9.1 months, and the remission rate of intracranial lesions was 67.6%, which is similar to the results of the data from our center (intracranial lesion remission rate of 82.2%) 24. Meanwhile, the efficacy of SIB-WBRT was also included in the study by Lu et al. There were 66 patients with brain metastases from non-small cell lung cancer (NSCLC) in the SIB-WBRT group in the study, and the 2-year iPFS rate of patients in this group was better than that of the other group, the WBRT group (49.3% vs. 34.5%, P = 0.041), and because the pathologic type of the whole group of patients was non-small cell lung cancer, so the median iPFS reached 22.3 months 25. In addition, the efficacy of SIB-WBRT was also reported in a study by a German scholar published in 2021, in which the SIB-WBRT group consisted of 62 patients, and the 1-year local control rate of tumors in this group was 98.0%, with a median iPFS of 13.5 months, which is similar to, but slightly superior to, our results overall 26. The data from the above studies had similar results to the data reported in this study. It suggests that SIB-WBRT is efficacious and reliable, and has great promise for future development as an emerging technology.

SIB-WBRT may lead to the development of radionecrosis. Regarding side effects, in studies related to SIB-WBRT, it has been reported that patients who had been treated with SIB-WBRT developed radionecrosis at the follow-up review, but this did not occur in our group of patients 26. It may be due to the small sample size of our patients, which was not large enough to observe the occurrence of radionecrosis or there were patients who had developed radionecrosis but were excluded from this study at the inclusion stage due not to subsequent follow-up in our hospital. In addition, it is difficult to determine radionecrosis by non-surgical methods, it is difficult to differentiate from tumor progression and often requires multiple reviews or functional imaging to assist in the determination, but this information was lacking during our follow-up. Some patients had only one review result, and in reality, their radionecrosis occurred but was not captured by imaging at follow-up.

Studies on the prolongation of patient survival with SIB-WBRT have been less well reported, and no large-scale clinical trials or randomized controlled trials have been able to definitively answer this question. However, a number of small-sample studies and observational studies have provided some limited evidence to support the idea that SIB-WBRT may have a positive impact on patient survival. A study from Geneva in 2010 included 53 patients with brain metastases from lung cancer, and univariate analyses showed that the improvement in OS was significantly correlated with the total dose (< or = 39Gy vs > 39Gy; p<0.01) 19. Scholars from Germany, in their retrospective study published in 2020 even observed a significant difference in survival between the two groups of patients after treatment with SIB-WBRT versus other radiotherapy modalities (9.9 vs. 6.2 months; P=0.001) and patients in the SIB-WBRT group achieved a median survival of 9.9 months, which the authors suggested may be due to the fact that this group of patients underwent head radiotherapy in conjunction with the benefit from systemic therapy 26. However, this same author also mentions the 2004 article from Pennsylvania, USA, which reported that an elevated tumor irradiated dose also resulted in a benefit to patient survival, so the positive impact of SIB-WBRT on patients is not completely ruled out 20. A median survival of 15.7 months was also observed in the patients in this study, but due to the lack of a control group, this could not be further analyzed to determine this.

### Limitations

The results of this study should be interpreted in the context of the following limitations. First, the entire study was lack of a standardized control group. Second, there was some missing data in patients' medical records, and there was irregular follow-up for part of the patients, which may have had an impact on treatment evaluation. Third, there was a lack of relevant data on cognitive changes after SIB-WBRT treatment.

## Conclusion

In patients with brain metastases receiving SIB-WBRT treatment, iPFS, local control, and new onset reached clinically acceptable levels. Additionally, the number of brain metastases in patients before treatment may be related to iPFS.

## Figures and Tables

**Table 1:** Baseline clinical characteristics of patients with brain metastases.

Characteristic (107)	Number of patients
**Age(years)**	(59.3±9.7)
<60	48(44.9%)
≥60	59(55.1%)
**Sex**	
Male	63(58.9%)
Female	44(41.1%)
**Histology**	
Small cell lung cancer	41(38.3%)
Non-small cell lung cancer	66(61.7%)
**Extracranial disease status**	
Controlled	4(3.7%)
Uncontrolled	103(96.3%)
**Targeted therapy**	
Yes	52(48.6%)
No	55(51.4%)
**GTV volume(cm^3^)**	
<8.4	53(49.5%)
≥8.4	54(50.5%)
**GTV number**	
>10	18(16.8%)
≤10	89(83.2%)

**Table 2:** Follow-up Results.

Totally (107)		SIB-WBRT
**iPFS**		
	iPFS(%)	49.5(53/107)
	Time to iPFS Failure(range) (mo)	0.6-45.7
	Median(mo)	13.4(95%: 4.2-22.6)
	iPFS at 6 months (%)	68.0(95%CI: 57.4-78.6)
	iPFS at 12 months (%)	50.8(95%CI: 38.3-63.3)
**Overall Survival**		
	OS(%)	29.9(32/107)
	OS of SCLC (%)	31.7(13/41)
	OS of NSCLC (%)	28.8(19/66)
	Follow-up time (months)	1.3-90.4
	Median follow up time (months)	13.3
	Follow up time >12 months (%)	53.3(57/107)
	Median (range) (mo)	15.7(95%CI: 10.4-21.1)
	Overall Survival at 6 months (%)	79.3(95%CI: 71.5-87.1)
	Overall Survival at 12 months (%)	58.9(95%CI: 49.3-68.5)
	Overall Survival at 24 months (%)	35.9(95%CI: 25.9-45.9)
**Local Control**		
	Local Control (%)	82.2(638/776)
	Time to Local Failure(range) (mo)	0.9-46.6
	Median (mo)	37.6(95%CI: 28.3-46.8)
	Local Control at 6 months (%)	84.3(95%CI: 80.6-88.0)
	Local Control at 12 months (%)	73.3(95%CI: 68.2-78.4)
**New Lesions**		
	New Cranial Lesion(s) (%)	39.3(42/107)
	Time to New Lesion(s) (range) (mo)	0.7-45.7
	Median (mo)	17.4(95%CI: 14.1-20.8)
	No New Lesion & Survival at 6 months (%)	74.5(95%CI: 64.5-84.5)
	No New Lesion & Survival at 12 months (%)	61.5(95%CI: 49.0-74.0)

**Table 3:** Univariate and multivariate analysis of iPFS in patients with brain metastases

Characteristics	Univariate analysis		Multivariate analysis	
	Hazard ratio(95%CI)	*P* value	Hazard ratio(95%CI)	*P* value
Sex		0.740		0.984
Male	Reference		Reference	
Female	0.9(0.5-1.6)		1.0(0.5-2.1)	
Age	1.0(0.989-1.1)	0.219	1.0(0.991-1.1)	0.161
Type		0.371		0.764
Non-small cell lung cancer	Reference		Reference	
Small cell lung cancer	1.3(0.7-2.5)		1.1(0.5-2.7)	
GTV volume	1.0(0.99-1.03)	0.330	1.1(0.9-1.3)	0.268
GTV number		0.075		0.043
≤10	0.5(0.2-1.1)		0.4(0.2-0.973)	
>10	Reference		Reference	
Extracranial disease status		0.399		0.258
Uncontrolled	Reference		Reference	
Controlled	1.7(0.5-5.5)		2.1(0.6-7.3)	
Targeted therapy		0.437		0.700
Yes	Reference		Reference	
NO	0.8(0.5-1.4)		0.9(0.4-2.0)	
GTV proportion	/	/	/	/

**Table 4:** Compare baseline data of patients with and without intracranial progression

Characteristic	Number of not intracranial progression patients (54)	Number of intracranial progression patients (53)	P* value
**Age(years)**			0.847
<60	25(46.3%)	23(43.4%)	
≥60	29(53.7%)	30(56.6%)	
**Sex**			0.697
Male	33(61.1%)	30(56.6%)	
Female	21(38.9%)	23(43.4%)	
**Histology**			0.234
Small cell lung cancer	24(44.4%)	17(32.1%)	
Non-small cell lung cancer	30(55.6%)	36(67.9%)	
**Extracranial disease status**			0.363
Controlled	1(1.9%)	3(5.7%)	
Uncontrolled	53(98.1%)	50(94.3%)	
**Targeted therapy**			0.123
Yes	22(40.7%)	30(56.6%)	
No	32(59.3%)	23(43.4%)	
**GTV volume(cm^3^)**			0.848
<8.4	26(48.1%)	27(50.9%)	
≥8.4	28(51.9%)	26(49.1%)	
**GTV number**			0.797
>10	10(18.5%)	8(15.1%)	
≤10	44(81.5%)	45(84.9%)	

*Fisher's exact test
